# Interaction Patterns between Wildlife and Cattle Reveal Opportunities for Mycobacteria Transmission in Farms from North-Eastern Atlantic Iberian Peninsula

**DOI:** 10.3390/ani11082364

**Published:** 2021-08-10

**Authors:** Lucía Varela-Castro, Iker A. Sevilla, Ariane Payne, Emmanuelle Gilot-Fromont, Marta Barral

**Affiliations:** 1 Animal Health Department, NEIKER-Basque Institute for Agricultural Research and Development, Basque Research and Technology Alliance (BRTA), Parque Científico y Tecnológico de Bizkaia, P812, E-48160 Derio, Spain; isevilla@neiker.eus (I.A.S.); mbarral@neiker.eus (M.B.); 2 Unité Sanitaire de la Faune, OFB/DGDPCE/DRAS, 45100 Orléans, France; ariane.payne@ofb.gouv.fr; 3 UMR 5558 LBBE, VetAgro Sup, Université de Lyon, 1 Avenue Bourgelat, F-69280 Marcy-l’Etoile, France; emmanuelle.gilotfromont@vetagro-sup.fr

**Keywords:** camera-traps, interactions, wildlife-livestock interface, tuberculosis, non-tuberculous mycobacteria

## Abstract

**Simple Summary:**

Mycobacteria can cause medically and socio-economically significant diseases, including several non-tuberculous infections and tuberculosis, and are considered a One Health challenge for their impact on public and animal health. These microorganisms are maintained and shared between the environment, domestic and wild animals, and humans. The aim of this research was to characterize the interactions that take place between several wild mammals and cattle through camera-trapping in order to provide insights into the dynamics of mycobacteria transmission opportunities in the environment of cattle farms located in Atlantic habitats from northern Iberian Peninsula. Camera traps were set during a one-year period in three cattle farms and visits of six wild species were modelled. We demonstrated that cross-species mycobacteria transmission, if occurring, would be mainly maintained through indirect interactions and most likely occur in pastures. In contrast to previous studies, wildlife visits were abundant but brief, and food and water resources did not attract wild animals. We suggest that badger latrines might act as aggregation points and sources of exposure to mycobacteria for badgers, wild boars, foxes, and cattle. This knowledge can contribute to designing and implementing effective measures aimed at controlling the spread of mycobacterioses in the environment–wild–domestic–human interface.

**Abstract:**

Interactions taking place between sympatric wildlife and livestock may contribute to interspecies transmission of the *Mycobacterium tuberculosis* complex or non-tuberculous mycobacteria, leading to the spread of relevant mycobacterioses or to interferences with the diagnosis of tuberculosis. The aim of this study was to characterize the spatiotemporal patterns of interactions between wildlife and cattle in a low bovine tuberculosis prevalence Atlantic region. Camera traps were set during a one-year period in cattle farms with a history of tuberculosis and/or non-tuberculous mycobacterioses. The frequency and duration of wildlife visits, and the number of individuals per visit, were analysed through generalized linear mixed models. The seasons, type of place, type of point, and period of the day were the explanatory variables. A total of 1293 visits were recorded during 2741 days of camera observation. Only 23 visits showed direct contacts with cattle, suggesting that mycobacteria transmission at the wildlife–livestock interface would occur mainly through indirect interactions. Cattle pastures represented the most appropriate habitat for interspecies transmission of mycobacteria, and badgers’ latrines appear to be a potential hotspot for mycobacteria circulation between badgers, wild boars, foxes, and cattle. According to both previous epidemiological information and the interaction patterns observed, wild boars, badgers, foxes, and small rodents are the species or group most often in contact with livestock, and thus may be the most involved in the epidemiology of mycobacterioses in the wildlife–livestock interface in this area.

## 1. Introduction

Multi-host pathogens are often of wide concern because of the complexity that entails their control [1]. This control may become harder to manage when wild species are involved in their maintenance and transmission and even more difficult when poor or lacking farm biosecurity measures enable the occurrence of interactions between livestock and wildlife. In order to improve biosecurity, it is necessary to identify the places, moments, and circumstances that entail highest risk. Generally speaking, the rate of interactions between species tends to increase when scarce water or food sources are shared by domestic and wild species, such as in Mediterranean ecosystems, due to a high spatial and/or temporal overlap between them [2,3] and so does the probability of pathogen spread and transmission. Indirect transmission, more likely than direct transmission, tends to be involved across a community of host species [4]. However, when several host species are involved in the transmission of the same pathogen, it is crucial to identify the most important epidemiological connections between species and where/when these connections occur. Understanding interactions that can potentially lead to pathogen transmission at the wildlife-livestock interface is therefore a key for the implementation of appropriate disease control strategies in a multi-host system. However, this is often difficult to assess.

Animal tuberculosis (TB) is a worldwide zoonotic disease caused mainly by *Mycobacterium bovis* and other mycobacteria belonging to the *Mycobacterium tuberculosis* complex (MTC). Although cattle are considered its main and most well-studied host, *M. bovis* represents the perfect example of a multi-host pathogen with a complex and diverse spectrum of both domestic and wild hosts. In fact, a recent study has demonstrated that TB systems in some regions of Europe are dominated by non-bovine domestic and wild species [5]. *M. bovis* survival in the environment is highly variable according to environmental conditions but may last for several months [6], enhancing the likelihood of interspecies transmission within shared habitats (mainly through indirect contacts) [2,3]. On the other hand, the emerging prevalence of non-tuberculous mycobacteria (NTM) has become a matter of concern [7], even in countries reporting a low TB incidence [8]. Some of these NTM are associated with opportunistic or major mycobacterioses affecting humans and several domestic and wild species, as well as with interferences in the diagnosis of bovine TB [9]. NTM are widely distributed in a broad variety of aquatic and terrestrial environments [10]. Some species of veterinary relevance, such as *Mycobacterium avium* subsp. *paratuberculosis*, are able to persist in the environment for long periods [11].

In the Iberian Peninsula, multiple domestic and wild hosts are implicated in the epidemiology of animal TB. Among domestic species, cattle is still considered the main reservoir [12], despite the fact that other livestock can also play this epidemiological role (e.g., goats [13], sheep [14], and pigs) [15]. Although Spain is far from being considered officially TB free, the herd-level prevalence in cattle has been greatly reduced since the introduction of the National Eradication Program in 1987. In Atlantic regions in particular, this prevalence has been kept below one per cent [16] for the last twelve years. However, eradication has not been accomplished yet. In spite of the absence of a mandatory NTM surveillance program, NTM-infected cattle have also been detected in these regions during the national TB eradication campaigns among cattle showing false positive reactions to the tuberculin skin test [17,18]. The interactions between cattle and competent cohabiting wild hosts could contribute to this epidemiological picture of Atlantic Iberian Peninsula. There, the European badger (*Meles meles*) has been described as a potential wild reservoir of TB [19,20]. Furthermore, occasional TB cases have been detected in red deer (*Cervus elaphus*), and wild boar (*Sus scrofa*) seems to be implicated in the epidemiology of the disease (its role still being under debate) [17,21,22]. Besides, several species of NTM have been detected in these three wild species as well as in roe deer (*Capreolus capreolus*), wood mice (*Apodemus sylvaticus*), fox (*Vulpes vulpes*), and other carnivores such as the stone marten (*Martes foina*) and the mink (Varela-Castro, unpublished data) [17,18,23]. Before designing and implementing strategies aimed at reducing pathogens transmission between wild and domestic animals, deepening our current understanding of wild-domestic interaction dynamics is necessary. Among the current tools available for this purpose, camera trapping is a non-invasive technique useful for the assessment of a broad variety of ecological phenomena [24,25,26,27], which can be helpful to delve into disease transmission mechanisms.

The aims of the present research were (1) to study through camera trapping the nature of interactions (direct or indirect, frequency, duration and number of animals per wildlife visit, and observed behaviours) between cattle and wild mammal species from the Basque Country, a low bovine TB prevalence Atlantic region, and (2) to investigate whether these interactions may vary according to season, period of the day, places and points sampled. The results will provide useful information for assessing the risk of transmission of mycobacteria that could help in designing potential control strategies adapted to this specific scenario.

## 2. Materials and Methods

### 2.1. Study Area

This study was carried out in the Basque Country, northern Iberian Peninsula, where the annual prevalence of TB among cattle herds has been less than one per cent for the last 17 years and kept below 0.1% since 2017 [16]. According to official censuses from 2018 [28], there are 134,611 cattle in 4703 farms. These animals graze in the pastures regardless of the management system. Even when managed under an intensive production system, enclosures are open to the field and no biosafety measures such as fencing are always implemented. Therefore, cattle may share the pastures with cohabiting wildlife. Traditional husbandry practices are still maintained by some farmers in the Basque Country, being communal pastures shared by cattle and other domestic species such as horses and sheep, during the summer. MTC infection among wild mammals from this region has been detected in wild boar (1.12%) and red deer (2.40%) [22]. Several species of NTM able to infect cattle and interfere with the diagnosis of bovine tuberculosis have been also detected in wood mice [18] and other wild species from the study area (Varela-Castro, unpublished data: wild boar, red deer, roe deer, badgers, foxes and stone martens).

**Figure 1:**
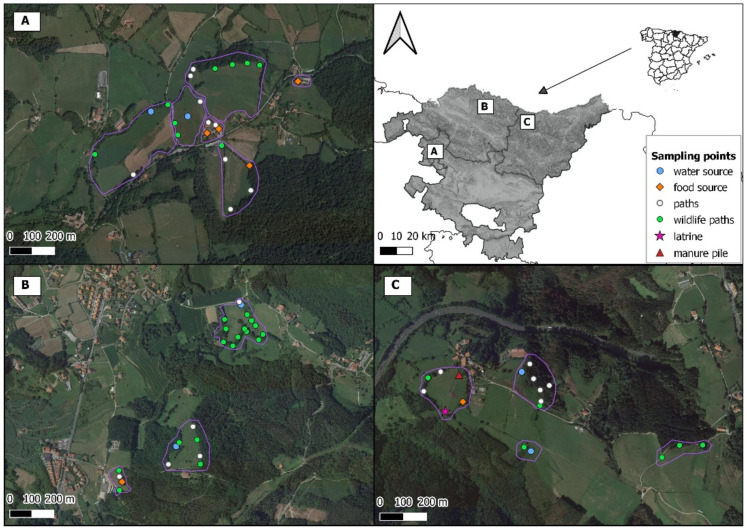
Field sampling design. The map shows the location of the studied farms (**A**–**C**) within the Basque Country (northern Iberian Peninsula). The spatial distribution of the sampling points recorded within farms (**A**–**C**) is displayed on the satellite photographs (**A**–**C**), respectively (see legend for sampling point description). Purple lines surround the sites (1 to 13) included in the models as a random factor.

Three cattle farms located in the municipalities of Kexaa, Kortezubi and Deba (named A, B and C, respectively (see Figure 1)) were selected to represent farms where TB and other mycobacterioses, mainly provoked by *M. avium* subsp. *paratuberculosis* and/or *Mycobacterium avium* subsp. *avium*, have been recently diagnosed. These farms hosted tuberculin skin test-reactor cattle that were subsequently confirmed as *M. bovis*-infected or as false positive cases. Almost half of the MTC-positive cattle of the last ten years in the Basque Country were detected in farms A and C and NTM were also detected in these farms, while in farm B only *M. avium* subsp. *avium* was detected [18]. Farms A and B are dairy farms and farm C is a fighting bull farm. All three farms follow a free-range system. Cattle from farm A can either graze in the pastures or stay indoors, since facilities are open all year long. Facilities from farm B are completely closed and cattle are kept indoors during autumn and winter. Bulls of farm C are always kept outdoors. There are no other domestic species in these farms that could be important in terms of MTC transmission.

### 2.2. Camera Trap Survey

During a one-year period (January to November 2017), a total of twenty-three infrared motion-triggered camera traps (CTs) (Trophy Cam HD Aggressor, Bushnell, Overland Park, KS, USA) were used for the detection of wild mammal visits in different places of the farms while cattle were present (either in the field or inside facilities connected with outdoors). The field design comprised a two-week sampling period per farm and season except for farm B, where cattle are kept in closed facilities with no contact with outdoors during autumn and winter; thus, no sampling periods were recorded for those seasons. Overall, 10 sampling periods were recorded. CTs owned movement detection up to 25 m and a response time of 0.2 s. They were programmed to work day and night, recording 10 s videos each time a movement was detected with a triggered interval of 5 s. Date and time were displayed for each video. CTs were tied on trees, spikes, fences, or walls at ≈50 cm above the ground or up to 150–200 cm with a downward inclination, depending on the sampling point. When needed, branches that fell in the field of vision were removed. There was no overlap between the CTs’ field of view.

The sampled places were cattle pastures, bushy edges between pastures, farm buildings, and a pine forest. In each place CTs were set in one to several points that could be *a priori* attractive for some wild species, such as water or food sources, a badger latrine and a manure pile, as well as in points that could potentially indicate the presence of wildlife, such as wildlife paths or paths that could be used by both cattle and wild species (see Figure 1). Water sources were all located outdoors and could be either a stream, a pond or cattle troughs settled with a certain height but surrounded by a flooded ground. Food sources were located indoors and outdoors. Those located indoors could be cattle feeders settled on the ground or feed-storages with some spillage of grain, while those located outdoors were piles of straw or hay delivered on the ground or a hazelnut trees plantation. Because of husbandry practices such as the rotation of herds among different pastures, the number of cameras varied between sampling periods and some points were not recorded during all the sampling periods of each farm. Overall, we sampled 67 points located in 17 places (Table 1). Due to the unbalanced availability among sampling places and points in the study area, pastures and wildlife paths were the type of place and point of the survey with longer surveillance time (Table 1). All videos were checked for species identification. If a wild mammal was detected, the number of animals, their behaviour and the duration of the visit was also registered.

### 2.3. Variables Definition

Since some CTs were located relatively close to each other, their observations could be non-independent. In each farm, we thus defined three to six sites, a “site” being a spatial unit corresponding to either a farm building or a pasture, including its bushy edges and the forest when present (see Figure 1). Each site (*n* = 13) was thus considered an independent area from other sites from the same farm, while the non-independence of observations within a site was accounted for in the analysis (see below). Distances between sampling points situated within a site varied among sites (range = 16 to 393 m). 

We defined a “session” as a continuous period of monitoring on the same sampling point with the same camera. Although sessions were planned to last two weeks, some of them terminated earlier due to cattle moving CTs, thefts and unexpected battery depletion. For this reason, the duration (in hours) of each session was taken into account for subsequent analyses. We defined independent visits as (1) consecutive videos of individuals of different species; (2) consecutive videos of individuals of the same species more than 30 min apart; or (3) non-consecutive videos of a different or same species [29]. The number of visits per wild species, their duration (interval between the time displayed at the beginning of the first video and at the end of the last video included in the same visit, in minutes), and the number of animals per visit were the dependent variables. Animals could not always be individually identified, so the maximum number of individuals seen simultaneously in any of the videos of each visit was recorded. A direct interaction was defined as the simultaneous presence of cattle and at least one wild mammal on the same video. All other visits were considered as indirect interactions with cattle, since all points included areas used by cattle. Explanatory variables were the season (spring: April–June, summer: July–September, autumn: October–November, and winter: January–March), the period of the day (dawn, day, night and sunset, being the time slots determined according to the season where visits were observed), the place (pasture, farm building, forest, and edge) and the sampling point (water source, food source, manure, latrine, path, and wildlife path). 

### 2.4. Statistical Analysis

The observed behaviours were classified focusing on those that could represent a risk of mycobacteria acquisition or excretion (Table 2). When none of these behaviours was observed, animals were considered as “moving through”. If more than one behaviour were detected during a visit, either by one or more individuals, they were all recorded, except for “moving through” [30]. The percentage of occurrence of the different behaviours was calculated for each species. Then, the frequencies of wildlife visits were described, for each species, in terms of means and standard errors (SEs) by computing the number of visits per month, based on the observations obtained for each session. The means and SEs were also computed for the duration of the visits and the number of individuals per visit. Afterwards, a description of the direct interactions between each species and cattle was performed.

Then, generalized linear mixed models (GLMMs) were used to analyse how the number of visits per each species, their duration and the number of individuals varied among seasons, periods of the day, type of place and type of point, using the farms (A, B and C) and the sites (1–13) within each farm as random effects in order to take into account the likely dependence of wildlife visits within farms and each site of every farm. For the number of visits, a model adjusted to a Poisson distribution was used and, in order to consider the sampling effort of the sessions, the logarithm of the number of surveillance hours per session was included in the model as an offset. For the number of animals and the duration of visits, models adjusted to Poisson and Gamma distributions were used respectively. A total of 18 models were initially fit, one per response variable and species. For each one, a maximal model including all the variables was first created. Hereafter, the dredge function of R software was used to generate a selection table of models with combinations of the fixed variables originally included in the maximal model. The selection of the best combination was made following the parsimony principle [31]: among models that had similar AIC values (delta < 2), the one with fewest parameters was selected.

Finally, we used the overdisp.glmer function of R in order to check whether overdispersion was still present in the residuals of the selected Poisson models [32]. Nakagawa and Schielzeth R-squared were used to determine the variability explained by the fixed and random parts of the selected models (using the r.squaredGLMM function of R software). All of the statistical analyses were performed using the R 4.0.0 software [33]. The data sets employed for the statistical analyses are submitted as Supplementary Material: Tables S1 and S2.

## 3. Results

### 3.1. Data Collected from the Field Samplings

Data were recorded during 2741 camera days (i.e., data obtained from a given camera over a given day) distributed into 180 sessions (mean duration ± standard error: 271.76 h ± 7.73). A total of 127,091 videos were recorded. Among them, 48,976 involved only cattle, 1329 other domestic species (cats, dogs, and horses), 4942 birds, 2320 wild mammals, and 4 reptiles. In 71 videos, it was not possible to identify the species. Wild mammal videos involved wild boar, roe deer, badger, fox, other carnivores (hereafter OC group, which includes genet (*Genetta genetta*), stone martens and pine martens (*Martes martes*)), small rodents (mouse-like), hedgehogs (*Erinaceus europaeus*), squirrels (*Sciurus vulgaris*), and bats. After excluding those species without previous epidemiological data on mycobacterial infection in the study area (hedgehogs, squirrels and bats), 2182 videos of wild mammals were retained for the analyses.

A total of 1293 visits by wild species of interest were registered, each visit being recorded by 1 to 33 videos. All species visited the farms during all seasons. Pastures and wildlife paths received the highest number of visits (Table 1). Since the observed species were mainly nocturnal, most of the visits (85%), including direct contacts with cattle, took place at night. Visits occurred in 64 out of the 67 sampling points. The three points that did not receive any visits were food sources located inside a farm building (2 points) and in a pasture (1 point). Wild boar, fox and small rodents were the only visitors of farm buildings (Table 3).

### 3.2. Frequency and Characterization of Visits Per Species

**Figure 2:**
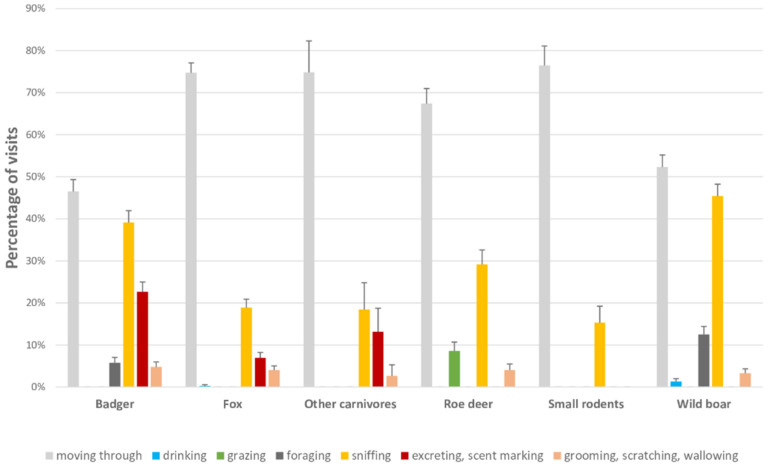
Percentage of the occurrence of each behaviour exhibited per species.

Figure 2 shows the proportion of occurrence of the behaviours exhibited per species. The most frequent behaviour was moving through (60% of the visits), followed by sniffing (31%), being both behaviours displayed by all species. Table 3 describes wild mammal visits in terms of frequency, number of individuals per visit, and duration of visits. The frequency of visits was highest for foxes, followed by badgers, wild boar, roe deer, small rodents, and the OC group. Small rodents were the group that showed longest visits on average (5.12 min ± 1.68), while the OC group showed the shortest on average (0.47 min ± 0.13). The species that showed up in more numerous groups was the wild boar (2.57 individuals ± 0.10, up to 10 individuals), while the rest of the species showed mainly solitary incursions (82% of visits performed by a single individual) or appeared, punctually, in small groups (up to four badgers, up to three roe deer, foxes, and small rodents). Even though visits longer than half an hour occurred sporadically (1% of the visits) except for the OC group, short visits (less than 5 min) were predominant (93% of the visits). Twenty-three direct contacts with cattle were recorded (see Table 4). Thus, the other 1270 visits were considered as indirect interactions. The fox was the species which showed most direct contacts with cattle (eight), followed by small rodents (six) and wild boar (six), badger (two) and roe deer (one). No direct interaction was recorded for the OC group. More than half of these direct contacts took place during autumn (13/23) and within pastures (17/23), being more frequent in wildlife paths (9/23). Those that took place in farm buildings were mostly between small rodents and cattle (4/5). Even though the most common behaviour recorded during the visits was moving through, when a direct interaction took place, wild animals showed other behaviours such as sniffing, scent marking or foraging, except for small rodents (Table 4).

Table 5 shows the outputs of the models selected to explain the frequency of visits, the number of individuals per visit, and the duration of visits for each wild mammal. Models related to the number of individuals could only be fit for wild boar data, due to the quasi-absence of variability for other species. The overdispersion of residuals was limited for all Poisson models, ranging from 0.6 to 3.03. The random effects (farms and sites within farms) accounted for 0 to 91.5% of the variations.

#### 3.2.1. Badger

No badger visit was recorded during the day, inside farm buildings, or in food sources. The two direct interactions with cattle were both observed in the latrine (Table 4). Pastures were the place where badger visits were most frequent and longest. The frequency of visits was also significantly higher in winter than in summer (OR = 2.49, 95% CI: 1.75–3.54). Moreover, visits were significantly more frequent in the badger latrine than in wildlife paths (OR = 3.81, 95% CI: 1.97–7.35), but significantly less frequent in paths (OR = 0.46, 95% CI: 0.31–0.68) or in the manure pile (OR = 0.04, 95% CI: 0.01–0.31) than in wildlife paths. The duration of visits was also significantly shorter in paths (OR = 0.41, 95% CI: 0.25–0.70) compared to wildlife paths (Table 5).

#### 3.2.2. Wild Boar

No diurnal visit was recorded. Apart from one case that was recorded in a farm building, direct interactions with cattle took place in pastures (5/6) (Table 4), including one in the latrine. The frequency of visits was significantly higher in autumn (OR = 1.57, 95% CI: 1.13–2.18) and spring (OR = 1.38, 95% CI: 1.02–1.88) compared to summer, while it was significantly lower during winter (OR = 0.39, 95% CI: 0.23–0.67). The same tendency was also observed for the number of animals per visit, being significantly higher in autumn compared to summer (OR = 1.52, 95% CI: 1.20–1.94). However, the visits were significantly longer in winter (OR = 3.41, 95% CI: 1.54–7.57). Wild boar visits were significantly more frequent in the badger’s latrine (OR = 9.07, 95% CI: 4.47–18.42) and significantly less frequent in food sources (OR = 0.34, 95% CI: 0.13–0.94). Lastly, wild boars were significantly less numerous during the dawn than at night (OR = 0.59, 95% CI: 0.38–0.92) (Table 5).

#### 3.2.3. Roe Deer

No visits were recorded in farm buildings, in the manure pile or in the latrine. The only direct interaction with cattle recorded took place in summer, during the day and in a wildlife path located in pastures (Table 4). Visits were significantly less frequent during spring (OR = 0.30, 95% CI: 0.21–0.44) and shorter during spring (OR = 0.10, 95% CI: 0.06–0.16) and autumn (OR = 0.28, 95% CI: 0.10–0.81), if compared with summer. Visits were significantly longer in food sources than in wildlife paths (OR = 22.15, 95% CI: 4.46–110.05) (Table 5).

#### 3.2.4. Fox

Foxes were seen at all periods of the day, type of places and type of points. All direct interactions with cattle but two that were recorded in the latrine and in a path took place in wildlife paths (6/8) (Table 4). Compared to wildlife paths, visits were significantly more frequent in badger’s latrine (OR = 2.79, 95% CI: 1.42–5.49) and less frequent in food (OR = 0.15, 95% CI: 0.05–0.42) or water sources (OR = 0.62, 95% CI: 0.42–0.93). Water sources received shorter visits (OR = 0.32, 95% CI: 0.13–0.83) than wildlife paths did. In comparison to summer, these were significantly longer in spring (OR = 2.02, 95% CI: 1.10–3.68) and shorter in autumn (OR = 0.44, 95% CI: 0.20–0.97). Moreover, they were significantly longer during the day than during the night (OR = 3.76, 95% CI: 1.61–8.78), and shorter in edges than in pastures (OR = 0.38, 95% CI: 0.15–0.97) (Table 5). 

#### 3.2.5. Other Carnivores

Most of these visits were performed by genets (33/38), even though stone martens (3/38) and martens (2/38) could be sporadically observed. All visits by these species were recorded in the pastures and their edges. No visit was recorded in food sources, in the latrine, or in the manure pile. Visits were significantly less frequent (OR = 0.19, 95% CI: 0.06–0.60) and shorter (OR = 0.36, 95% CI: 0.20–0.66) in pastures than in edges, and less frequent in water sources than in paths (OR = 0.20, 95% CI: 0.06–0.67) (Table 5).

#### 3.2.6. Small Rodents

No visit was recorded during sunset, in the forest, in the manure pile, or in the latrine. Unlike the rest of the species, direct interactions between cattle and small rodents were more frequent inside farm buildings (4/6) (Table 4). The selected models showed that frequency and duration of visits were significantly lower in summer (OR = 0.03, 95% CI: 0.01–0.09), winter (OR = 0.11, 95% CI: 0.05–0.23) and spring (OR = 0.15, 95% CI: 0.08–0.29) compared to autumn. Their frequency was also lower in edges compared to pastures (OR = 0.33, 95% CI: 0.15–0.72) and in paths compared to wildlife paths (OR = 0.53, 95% CI: 0.31–0.92) (Table 5).

## 4. Discussion

### 4.1. Methodology

Camera trapping has proved to be a useful tool for studying interactions with a minimal disturbance to animals. However, failures in the detection due to intrinsic characteristics of the CTs (distance detection, response time), the position angle when hanging the devices, camera malfunctioning, and loss of battery power or adverse weather conditions may have led to an underestimation of the number and duration of visits and the number of individuals. Hence, our observations correspond to a minimum of what is actually occurring in these farms. On the other hand, due to husbandry practices and organization issues, there was an imbalance in the field sampling, since all points were not sampled at all seasons. This could have limited our ability to detect seasonal variations. Besides, the small sample size of some types of places and points (e.g., forest, latrine; see Table 1) has narrowed the information obtained from them and may be underrepresented.

### 4.2. Spatiotemporal Patterns of Wildlife-Cattle Interactions

Our observations confirmed that as well as in other regions [2,3,30,34], indirect interactions between wildlife and cattle can be considered more frequent than direct interactions. Different spatial and temporal patterns were observed depending on the species surveyed.

Winter was the most favourable season for badger visits to occur. This season is a period of food scarcity for this species, whose diet in the Basque Country is mainly based on earthworms and garden fruits [35]. However, badgers did not approach farm buildings or food sources, probably due to the fact that cattle resources (e.g., silage or hay) lack attractiveness for them. These findings are consistent with those previously reported in a medium density population area, where badgers clearly avoided farmyards [36] but differ with some British [37,38] and French studies [30], where high rates of building use were described. Resource availability and badger population density might account for these differences. In agreement with previous reports [39], pastures were the preferred place for badgers in our study area. Earthworm intake might explain the attractiveness of pastures, since their soft ground is suitable for foraging. The most attractive point for this mustelid was the badger latrine. Moreover, the few direct interactions recorded between cattle and badgers always took place in the latrine. For these reasons, this point might be considered as a potential hotspot for both indirect or direct interactions between these two species. However, in the absence of other latrines, it is not clear whether this high frequency of visits was due to the latrine itself or to another specific feature of this particular point.

The seasonal differences observed in the frequency and duration of wild boar visits, as well as in the number of individuals per visit, may be due to different factors. In winter, wild boar density is at its lowest and the duration of incursions may be longer when searching for food. However, farm food sources were the less attractive point for this species. This could be related to a higher availability of natural resources in the study area, at least compared to areas from southern Spain, where baited points turned out to be very attractive to wild boar [2]. As well as with badgers, wild boar visits were more frequent in the badger latrine, possibly due to an attraction effect of its characteristic scent. Thus, the latrine could also represent a significant point for wild boar to interact with other species.

Roe deer visits were most frequent and longest in summer, coinciding with the mating period of this species. Some of the activities during this period, such as the defence of the territory, the avoidance of dangerous fights and the chasing of females by bucks [40] might make them more visible. Actually, the only direct contact recorded between roe deer and cattle took place in pastures during summer and by day. Longer visits were observed in food sources than in other sampling points. Roe deer is mainly a browser, not a grazer [41], so they are not expected to use the same food resources as cattle. Indeed, all roe deer visits to food sources were recorded in the hazelnut trees plantation, which represents the only resource not interesting for cattle. 

As for badgers and wild boar, the badger latrine was the most visited point by foxes and also the scene of a direct interaction with cattle, which supports considering the badger latrine a potential hotspot for intra and interspecies interactions in our study area. Despite visiting all types of points, foxes showed less interest for food and water sources. This may reflect their interest in other food supplies, such as small mammals to prey on. Conversely to other species, fox visits were longer during the day because they spent a long time resting on the pastures. Lastly, visits were shorter in autumn and longer in spring compared to the summer. These findings could be also related to their rest times, which were longer during the warmest seasons.

OC group species turned out to be less often seen and always alone. Visits were most frequent and longest in the edges, which is consistent with their search for protection from predators and unfavourable weather conditions [42]. The majority of visits were performed by genets, and almost half of them were recorded in one specific path within the edge of one pasture. Genets spend most of their time resting in the same place [42]. Thus, the potential existence of a resting site close to this path might explain the output of the models. 

Although small rodent species could not be determined, in a previous study conducted in the same farms the wood mouse was the species most frequently captured [18]. Small rodents visits occurred more often and were longer in autumn than during other seasons, probably since this is a period when most rodent populations, such as the widely distributed wood mouse population, are at their maximal abundance [43]. These wild rodents typically move along field margins of farmlands and are known to be common in hedgerows [44], which might explain their preferences for pastures and wildlife paths from the study area.

### 4.3. Opportunities of Mycobacteria Transmission

Since indirect interactions were much more common than direct interactions, mycobacteria transmission at the wildlife-livestock interface, if occurring, would be mainly held through indirect interactions. In general, pastures represent the most appropriate place for interspecies transmission of mycobacteria in the study area. Our results suggest that badger latrines can be suitable places for both indirect and direct contacts at least between badgers, wild boar, foxes and cattle. During the visits to the latrine, individuals of these three wild species showed behaviours related to possible excretion of or exposure to pathogens such as sniffing or scent marking, where cattle was also seen sniffing or grazing. Consequently, these points could be considered potential hotspots for mycobacteria circulation in this habitat. However, a single latrine was found and recorded, and therefore, further studies are needed to confirm this hypothesis.

Some remarkable differences have been identified between this study and previous reports. In our study, whatever the wild species considered, the average duration of the visits was shorter (<5 min) than in studies from France [29,30]. For instance, the average duration of wild boar visits was significantly shorter in our study area (1.64 min) than in a bovine TB-infected area in France (14.5 min) [30]. The most common behaviour in our study area was “moving through”. Wild species mainly move around shared habitats with cattle, but resources such as water or food supplies do not act as aggregation points, conversely to the results of previous studies [2,29,30,45]. A higher availability of natural resources throughout the whole year may account for these differences. These findings, together with the absence of MTC-infected individuals among wildlife, except for wild boar, and the low TB prevalence reported for this wild ungulate and cattle from the Basque Country [22], suggest that the risk of MTC transmission between wild animals and cattle would be, overall, low. On the contrary, we suspect that a risk of indirect NTM transmission could be more feasible in the study area, since this group of mycobacteria have been detected in all species and the prevalence observed in some of them was significant.

Since interaction patterns and infection figures differed among wild species, some of them might be more involved than others in the epidemiology of mycobacterioses in the Basque Country. Thus, depending on the species and the situation, different control strategies could be implemented to maximize effectiveness. Foxes, badgers and wild boars were the species observed most frequently. Even though not considered as a TB reservoir in the Atlantic Iberian Peninsula, wild boar is the only species observed in this study and found to be infected with MTC in the study area, since red deer distribution is limited to a few settings that do not encompass these farms. Wild boar has shown an unexpectedly high MTC seroprevalence of 17% in this region [21] and, despite the low prevalence detected by culture (<2%) and the absence of animals with disseminated lesions/infection, a potential geographical link was found between spoligotypes identified in cattle and wild boar [22]. Furthermore, culture methods revealed a 9% prevalence of NTM in this species. Considering this information, the high frequency of visits and the high proportion of individuals per visit, wild boar could contribute to the dynamics of mycobacteria transmission in the Basque Country. Conversely, the badger is already considered a potential reservoir of TB in neighbouring Atlantic regions [19,20] but no infected individual was found in our study area [22]. However, a high prevalence of NTM infection (17%) was detected in this mustelid, as well as in other regions of northern Iberian Peninsula [23]. Since the ability of badger to transmit MTC is already confirmed, either as a TB maintenance host or as a bridge between other species through its latrines [46], the potential role of this carnivore in the epidemiology of mycobacterioses in the Basque Country should not be ruled out. MTC-infected foxes have been sporadically found in Spain [47] but not in the Basque Country, where 46 individuals were analysed throughout a 10-year survey [22]. The fox is currently considered a spill-over host of TB in Europe [48] (i.e., populations cannot maintain infection on the long-term, but may transmit it to other species), even though the prevalence reported in foxes ranged from 9% in four TB endemic areas of France [49] to 26% in Portugal [50,51]. In addition, the prevalence of NTM in foxes from the Basque Country (4.3%) was lower compared to badgers and wild boar. Nevertheless, the fox was the species most often observed and for which most direct contacts with cattle were recorded, so its behaviour could counteract its apparent irrelevance in the epidemiology of mycobacterioses. A study carried out earlier in the same three farms proved that small rodents such as *A. sylvaticus* can carry potentially pathogenic NTM with the ability to cross-react with TB diagnosis in cattle, reporting an overall prevalence of 6.5% [18]. However, no species belonging to the MTC were detected. The scarce literature available on the epidemiology of natural *M*. *bovis* infection in small rodents suggests that these animals could be dead-end hosts (i.e., not able to transmit infection to other species [52,53,54]). However, the field vole (*Microtus agrestis*) is considered as a natural maintenance host for *M*. *microti*, a role that other small rodents like the wood mouse might play, maintaining the infection and spreading the bacteria through wounds inflicted to their predators or by indirect transmission through sputum, saliva or skin crusts [55,56]. These routes should not be ruled out for NTM transmission. Although most of the visits in our study were recorded in pastures and wildlife paths, small rodents were also observed inside the farm buildings and, conversely to the rest of the studied species, most of their direct contacts with cattle took place inside the enclosures. Roe deer visits were on average more frequent than those of small rodents and longer compared to badgers, wild boars and foxes. In the Basque Country, no cases of TB were detected [22] and the prevalence of NTM in roe deer was 4.70%. Like the fox, the roe deer has been considered a spill-over host, particularly in endemic areas [57]. However, TB cases in roe deer are reported even more sporadically [58]. The behaviour of this species during this study (mostly solitary, not observed close to farm buildings, preference for food sources disregarded by other species and almost no direct contacts with cattle) suggests that roe deer is unlikely to play any role in the epidemiology of TB in this low prevalence area. Accordingly, its relevance in the epidemiology of other mycobacterial infections seems to be limited. Finally, OC group could be considered the least threatening in terms of mycobacteria transmission risk in the study area. If we focus on the species observed in this study, *M. avium* was detected in one stone marten out of 18 specimens analysed in the Basque Country. Besides, to the best of our knowledge the only cases of MTC infection reported in Europe belonged to one stone marten and two genets from Portugal [51]. These epidemiological features, as well as the behaviour observed (lowest frequency and duration of visits that are always performed by one individual, no direct interaction with cattle and preference of edges over pastures) support our statement.

The findings of this study together with previous results in wildlife from the Basque Country and the low TB infection prevalence observed in cattle do not show a strong justification for intervention to reduce the risk of mycobacteria transmission at the wildlife–livestock interface. However, monitoring has proved to be an essential tool for defining the most appropriate measures if the situation changes. To reduce wild visits to farms, combined strategies rather than a single one would be more effective [59]. This study suggests that biosafety should particularly concern pastures, for example by activating electric fencing at night and at different heights, taking into account the anatomy of the species of interest. Furthermore, according to our results the presence of badger latrines inside pastures should be at least identified and hereafter reduced or cattle access to them should be avoided. These measures may reduce indirectly the presence of wild boar and foxes in these particular points as well. Because measures directed at minimizing contacts between cattle and small rodents are difficult to implement, rodent population control strategies may indirectly help to reduce these interactions. Notwithstanding, MTC and *M. leprae* put aside and in spite of a few recognized pathogens such as *M. avium* subsp. *paratuberculosis* and *M. avium* subsp. *avium* [60,61], the vast majority of NTM are generally regarded as environmental and ubiquitous or opportunistic pathogens at the most [62,63]. This fact makes NTM control even more challenging. Strategies to avoid exposure to these mycobacteria may rely more strongly on hygiene than on preventing contact between animals. Possible measures include avoidance of animal-driven farm environmental contamination, water contamination, biofilm formation, and surface spreading [63].

## 5. Conclusions

The results of the present study combined with the information derived from our previous epidemiological surveys suggest that four wild species or groups might be most involved in the epidemiology of mycobacterioses in the Basque Country: wild boar, badgers, foxes, and small rodents. Cattle pastures were the most frequently visited habitat and indirect interactions represent the most likely route for the potential transmission of mycobacteria between cattle and wild species. Conversely to previous studies, food and water sources did not attract wild species in this region, while badger latrines could have acted as aggregation points and as a source of mycobacteria exposure for badgers, wild boars, foxes and cattle. Further studies are first needed to confirm that interactions between wild species and cattle in pastures occur preferentially on the latrines. If so, their identification and management would be a key to avoid inter-species transmission on pastures in this area. Moreover, analysing interactions among these three wild species around latrines and in other interfaces (elsewhere than in farm environment) will be needed to completely understand mycobacteria transmission dynamics. In agreement with the low TB prevalence of the study area, the risk of MTC transmission at the wildlife-livestock interface is expected to be low. However, the risk of NTM interspecies transmission in the Basque Country is more likely than that of MTC, which could result in the maintenance and spread of potentially pathogenic mycobacteria that could also affect the tuberculin test specificity in cattle. The current quantification and qualification of connections among several hosts provides a valuable insight into the dynamics of transmission within the wildlife-livestock interface.

## Figures and Tables

**Table 1:** Number of visits, surveillance recording hours and sessions grouped by type of place and point.

Type of Place	Number Surveyed	Type of Point	Number Surveyed	Number of Surveillance Hours	Number of Sessions
Pasture (1070)	11	Water source (114)	6	4514.83	17
		Food source (10)	3	939.60	4
		Manure (12)	1	1343.82	4
		Latrine (54)	1	511.27	2
		Wildlife path (703)	30	20,115.23	77
		Path (177)	13	8590.40	30
Farm building (22)	2	Food source (8)	3	2564.35	11
		Path (16)	2	1144.52	7
Forest (61)	1	Wildlife path (12)	1	360.23	1
		Path (49)	2	2756.07	8
Edge (140)	3	Wildlife path (17)	1	1455.08	4
		Path (109)	3	3689.23	12
		Water source (14)	1	1027.97	3
Total	17		67	49,012.60	180

Numbers in brackets indicate the number of wild mammal visits.

**Table 2:** Description of behaviours observed among wild species.

Behavior	Description
Grazing (for roe deer)	Feeding from grass, plants or fruits from a surface
Foraging (for badger and wild boar)	Searching for food by digging the ground with the snout
Sniffing (for all species)	Smelling the ground to search for food or to explore a surface/object
Excreting (for all species)/Scent marking (for all carnivores)	Urinating or defecating. For carnivores, lifting the tail and approaching the pelvis to the ground
Grooming/Scratching/Wallowing (for all species)	Applying tongue or paws to parts of the body in repeated motions, shaking the body, scraping against a surface, rolling in a water point
Drinking (for all species)	Drinking from water sources
Moving through (for all species)	Passing through a sampling point without performing any of the aforementioned behaviours

**Table 3:** Description of wild mammal visits. Mean ± SE and range are shown for frequency of visits per month, visit duration, and number of individuals per visit.

	Badger (*n* = 315)	Wild Boar (*n* = 304)	Roe Deer (*n* = 175)	Fox (*n* = 376)	Other Carnivores (*n* = 38)	Small Rodents (*n* = 85)
Frequency of visits (all visits, number per month)	4.73 ± 0.61 0–46.67	4.41 ± 0.58 0–46.33	2.76 ± 0.64 0–83.72	5.69 ± 0.66 0–59.20	0.54 ± 0.16 0–19.66	1.85 ± 0.74 0–117.2
Frequency of visits in buildings only (number per month)	0	1.07 ± 0.76 0–12	0	1.30 ± 0.76 0–12.12	0	2.04 ± 2.04 0–36.76
Visit duration (min)	0.89 ± 0.19 0.17–31	1.64 ± 0.26 0.17–38	2.33 ± 0.60 0.17–56	1.31 ± 0.31 0.17–86	0.47 ± 0.13 0.17–4	5.12 ± 1.68 0.17–95
Number of individuals per visit	1.05 ± 0.01 1–4	2.57 ± 0.10 1–10	1.16 ± 0.03 1–3	1.04 ± 0.01 1–3	1 ± 0 1–1	1.08 ± 0.04 1–3

Numbers in brackets indicate the total number of visits.

**Table 4:** Description of direct contacts between wild mammals and cattle.

	Badger	Wild Boar	Roe Deer	Fox	Small Rodents
Number of direct interactions	2	6	1	8	6
Most frequent season	Autumn (2)	Autumn (5)	Summer	Summer (5)	Autumn (4)
Most frequent place	Pasture (2)	Pasture (5)	Pasture	Pasture (7)	Farm building (4)
Most frequent point	Latrine (2)	Manure (2)/Wildlife path (2)	Wildlife path	Wildlife path (6)	Path (4)
Behaviors observed	Sniffing/ scent marking	Sniffing/foraging/moving through	Sniffing	Moving through/sniffing	Moving through

Numbers in brackets indicate the number of direct contacts.

**Table 5:** Models selected per wild species and response variable. For each model, the table gives the percentage of variation explained by fixed and random parts of the model, and the OR, estimate and *p*-value of Wald test for each contrast between the reference level and the given level. The number of individuals was analyzed for wild boar only due to the quasi-absence of variability for other species.

Species	Response Variable	V.E by Fixed Part	V.E by Random Part	Fixed Effect	Level	OR (95% CI)	Estimate	*p*-Value
Badger	Frequency of visits	28.35%	40.94%	Season	Autumn	0.87 (0.59–1.26)	−0.14	0.454
				(ref: summer)	Winter	2.49 (1.75–3.54)	0.91	<0.001 ***
					Spring	1.16 (0.86–1.58)	0.15	0.325
				Place	Edge	0.56 (0.34–0.91)	−0.59	0.020 *
				(ref: pasture)	Forest	1.06 (0.53–2.11)	0.06	0.875
				Point	Latrine	3.81 (1.97–7.35)	1.34	<0.001 ***
				(ref: wildlife path)	Manure	0.04 (0.01–0.31)	−3.17	0.002 **
					Path	0.46 (0.31–0.68)	−0.78	<0.001 ***
					Water source	0.81 (0.54–1.20)	−0.21	0.287
	Duration of visits	1.03%	9.09%	Place	Edge	0.43 (0.20–0.89)	−0.85	0.024 *
				(ref: pasture)	Forest	0.35 (0.15–0.80)	−1.06	0.013 *
				Point	Latrine	0.43 (0.18–1.03)	−0.84	0.059
				(ref: wildlife path)	Manure	0.27 (0.02–3.04)	−1.29	0.292
					Path	0.41 (0.25–0.70)	−0.88	<0.001 ***
					Water source	1.60 (0.95–2.70)	0.47	0.079
Wild boar	Frequency of visits	29.08%	19.64%	Season	Autumn	1.57 (1.13–2.18)	0.45	0.007 **
				(ref: summer)	Winter	0.39 (0.23–0.67)	−0.94	<0.001 ***
					Spring	1.38 (1.02–1.88)	0.32	0.038 *
				Point	Food source	0.34 (0.13–0.94)	−1.07	0.037 *
				(ref: wildlife path)	Latrine	9.07 (4.47–18.42)	2.20	<0.001 ***
					Manure	1.30 (0.49–3.41)	0.26	0.599
					Path	0.77 (0.55–1.08)	−0.26	0.128
					Water source	1.23 (0.86–1.76)	0.21	0.260
	Number of animals	10.85%	0.60%	Season	Autumn	1.52 (1.20–1.94)	0.42	<0.001 ***
				(ref: summer)	Winter	0.70 (0.46–1.07)	−0.35	0.103
					Spring	1.04 (0.83–1.30)	0.04	0.746
				Period of the day	Dawn	0.70 (0.34–1.43)	−0.35	0.332
				(ref: night)	Sunset	0.59 (0.38–0.92)	−0.53	0.020 *
	Duration of visits	0.32%	10.88%	Season	Autumn	1.12 (0.60–2.07)	0.11	0.730
				(ref: summer)	Winter	3.41 (1.54–7.57)	1.23	0.003 **
					Spring	1.21 (0.71–2.06)	0.19	0.480
Roe deer	Frequency of visits	4.87%	91.50%	Season	Autumn	1.57 (0.83–2.97)	0.45	0.162
				(ref: summer)	Winter	0.54 (0.25–1.18)	−0.61	0.123
					Spring	0.30 (0.21–0.44)	−1.19	<0.001 ***
	Duration of visits	6.21%	20.20%	Season	Autumn	0.28 (0.10–0.81)	−1.26	0.020 *
				(ref: summer)	Winter	0.95 (0.33–2.72)	−0.05	0.925
					Spring	0.10 (0.06–0.16)	−2.32	<0.001 ***
				Point	Food source	22.15 (4.46–110.05)	3.10	<0.001 ***
				(ref: wildlife path)	Path	1.43 (0.59–3.46)	0.35	0.434
					Water source	1.85 (0.42–8.11)	0.62	0.413
Fox	Frequency of visits	28.42%	6.43%	Point	Food source	0.15 (0.05–0.42)	−1.89	<0.001 ***
				(ref: wildlife path)	Latrine	2.79 (1.42–5.49)	1.03	0.003 **
					Manure	0.48 (0.19–1.23)	−0.73	0.127
					Path	1.02 (0.79–1.32)	0.02	0.863
					Water source	0.62 (0.42–0.93)	−0.47	0.020 *
	Duration of visits	3.88%	3.89%	Season	Autumn	0.44 (0.20–0.97)	−0.83	0.042 *
				(ref: summer)	Winter	0.86 (0.43–1.73)	−0.15	0.670
					Spring	2.02 (1.10–3.68)	0.70	0.022 *
				Period of the day	Dawn	0.89 (0.25–3.19)	−0.12	0.859
				(ref: night)	Day	3.76 (1.61–8.78)	1.33	0.002 **
					Sunset	0.60 (0.25–1.44)	−0.51	0.254
				Place	Edge	0.38 (0.15–0.97)	−0.97	0.042 *
				(ref: pasture)	Farm building	1.48 (0.13–16.54)	0.39	0.750
					Forest	0.45 (0.16–1.25)	−0.80	0.125
				Point	Food source	2.22 (0.15–33.41)	0.80	0.563
				(ref: wildlife path)	Latrine	0.23 (0.04–1.34)	−1.47	0.103
					Manure	0.12 (0.01–1.03)	−2.13	0.053.
					Path	0.68 (0.33–1.41)	−0.39	0.301
					Water source	0.32 (0.13–0.83)	−1.13	0.019 *
Other carnivores	Frequency of visits	2.68%	3.58%	Place (ref: edge)	Pasture	0.19 (0.06–0.60)	−1.68	0.005 **
				Point	Water source	0.20 (0.06–0.67)	−1.61	0.009 **
				(ref: path)	Wildlife path	0.34 (0.11–1.02)	−1.08	0.054.
	Duration of visits	6.9%	0.00%	Place (ref: edge)	Pasture	0.36 (0.20–0.66)	−1.02	<0.001 ***
Small rodents	Frequency of visits	23.92%	18.23%	Season	Summer	0.03 (0.01–0.09)	−3.64	<0.001 ***
				(ref: autumn)	Winter	0.11 (0.05–0.23)	−2.19	<0.001 ***
					Spring	0.15 (0.08–0.29)	−1.91	<0.001 ***
				Place (ref: pasture)	Edge	0.33 (0.15–0.72)	−1.11	0.005 **
					Farm building	1.86 (0.16–21.88)	0.62	0.623
				Point	Food source	0.20 (0.03–1.13)	−1.63	0.068
				(ref: wildlife path)	Path	0.53 (0.31–0.92)	−0.63	0.023 *
					Water source	0.42 (0.17–1.04)	−0.86	0.060
	Duration of visits	11.08%	0.00%	Season	Summer	0.02 (0.00–0.14)	−3.74	<0.001 ***
				(ref: autumn)	Winter	0.04 (0.01–0.11)	−3.31	<0.001 ***
					Spring	0.02 (0.01–0.06)	−3.74	<0.001 ***

V.E = Variation explained. ref = reference level of the fixed effect. *: *p*-value ≤ 0.05. **: *p*-value ≤ 0.01. ***: *p*-value ≤ 0.001.

## Data Availability

The data presented in this study are contained within the Supplementary Materials Tables S1 and S2.

## Supplementary Materials

The following are available online at https://www.mdpi.com/article/10.3390/ani11082364/s1, Table S1: Sessions database; Table S2: Visits database.
